# Quality of life of homosexual males with genital warts: a qualitative study

**DOI:** 10.1186/1756-0500-3-280

**Published:** 2010-11-04

**Authors:** Gitte Lee Mortensen, Helle K Larsen

**Affiliations:** 1 AnthroConsult, Aarhus, Denmark; 2 Venereal Diseases Clinic, Bispebjerg Hospital, Copenhagen, Denmark

## Abstract

**Background:**

A recent qualitative study in Denmark showed that genital warts (GWs) can considerably lower the quality of life of heterosexual patients. In this follow-up study, we interviewed men having sex with men (MSM) suffering from GWs to obtain an in-depth understanding of their perception of GWs and determine the extent to which minority (homosexual) cultural issues affect these patients' experiences. Qualitative interviews with six MSM were performed using a semi-structured interview guide. Questions were formulated on the basis of the earlier qualitative study in heterosexual patients with GWs along with a literature review. Data were analysed using a medical anthropological approach.

**Findings:**

Many MSM worried about being stigmatised and excluded from the small homosexual *'scene'*, their key social group, thereby lowering their chances of finding sex and love. Most participants had suffered from GWs for several years which added to the negative psycho-sexual and social effects of the disease. Participants' fears of developing anal cancer were similar to those expressed about cervical cancer by females with GWs.

**Conclusions:**

Ano-genital human papillomavirus (HPV) infection is common and has a serious psychological and sexual impact among MSM. However, they do not benefit to the same extent as heterosexual men from the herd immunity effect of HPV vaccination of girls. The pathological profile and concerns specific to MSM should be addressed when communicating with these patients, and should be taken into account when considering HPV vaccination of boys.

## Background

Genital warts (GWs), one of the most common sexually transmitted diseases (STD), can significantly lower patients' quality of life (QoL). A qualitative study carried out among young heterosexual Danes [1] showed that patients with GWs worried about the uncertain timeline and perspectives of recovery, and that they were often depressed and had low self esteem. The patients' self-image as well as their social lives were affected by the stigmatisation caused by the disease. Patients found it difficult to detach themselves from their GWs because of the repeated treatments, the social manoeuvres required to conceal the disease and the negative effects on their love and sex lives, in particular [1]. These results are in line with published data pointing to a substantial need for more knowledge about GWs and improved doctor-patient communication [2-16].

Fifteen articles (in English) on the effects of GWs on the QoL of patients of both sexes were identified from a literature search using PubMed, Embase, Cinahl and Psycinfo [2-16]. Four of the studies focused on the effects of human papillomavirus (HPV) infection in men [4-7]. A review of the articles showed that the effects of having GWs on QoL were similar for men and women. However, women tended to worry more about their fertility, infecting a baby during childbirth and the development of cervical cancer for those who knew about HPV [8,9]. One study revealed that men more frequently reacted with denial, delayed seeking treatment and worried about pain [6]. Only one study focused on men having sex with men (MSM) and their knowledge of HPV [4].

Approximately 90% of GWs are caused by infection with HPV types 6 and 11 [17]. The quadrivalent HPV vaccine that was introduced in the Danish childhood immunisation programme in October 2008 contains HPV 6 and 11 virus-like particles as well HPV type 16 and 18 virus-like particles (high-risk HPV types). In Denmark, this vaccine is recommended for the prevention of cervical cancer in 12-year-old girls with a catch-up programme for 13-15 year-old girls. A herd immunity effect on heterosexual men is expected, but such effects for MSM would be expected to be more limited [18].

GWs in MSM are often located in the intra- or perianal region, and it has been reported that anal warts are twice as frequent as GWs in this population [19]. Like the cervical tract, the anal canal has a transformation zone between the columnar epithelium of the rectum and the squamous epithelium of the anus, which is more receptive to HPV infection than the mucosa and skin of the rest of the ano-genital region [20]. The treatment of intra-anal warts is often protracted and painful, and recurrence rates are high. The impact of treatment on the risk of HPV transmission is uncertain [21,22].

HPV infection is highly prevalent in men and even more so in the case of MSM [23-28]. In one study, HPV DNA was found in the anal canal in 57% of HIV-negative MSM, with 26% having high-risk HPV types and 26% having low-risk HPV types; co-infection is frequent [29]. Anal infection with multiple HPV types is associated with receptive anal intercourse and the number of sex partners [29,30]. High-risk HPV types are found in almost all anal cancers in homosexual men, suggesting that HPV infection is prerequisite for the development of these cancers [31,32]. Although anal HPV infections are frequent, anal cancers are still relatively rare in Denmark (0.5 per 100 000 person-years) suggesting that unidentified factors influence the progression from HPV infection to cancer. Nonetheless, incidence rates of anal cancer have increased over the last half century, and incidence rates among MSM are high compared with that of the general population. It is estimated that the prevalence of anal cancer among MSM is currently at least as high as the prevalence of cervical cancer before the introduction of cervical screening programmes [29]. Incidence rates are particularly high among HIV-positive MSM, in spite of treatment with HAART (highly active antiretroviral therapy) [32,33].

Despite the fact that MSM have a higher risk than heterosexual men of contracting GWs, are less likely to benefit from the herd immunity effect of the vaccination programme, and are more likely to develop anal cancer, very little data are available on GWs in MSM. The objective of the present study, the first of its kind in MSM, was to examine in depth the way GWs may affect the QoL in this population and if particular minority cultural issues were relevant to their experiences with this disease.

## Methods

The previous study of heterosexual patients with GWs was based on two qualitative group interviews with a total of ten participants, five women and five men [1]. Qualitative research methods are considered the most appropriate for examining patients' perceptions of a disease [34,35]. In the present follow-up study, we initially planned to carry out a similar group interview with five or six MSM, however, patients were too reluctant to discuss the subject in a group. Hence we conducted individual interviews and sought to determine the reasons for their reluctance with a semi-structured question guide (Table 1), developed using a previously-described approach [1].

**Table 1:** Interview guide

Interview stage	Aim	Question
Opening	Introduction of the participant	What is your name, age and how long have you been suffering from GWs?

Introductory question	Participant's reaction to the GWs diagnosis	1. How did you react when you heard that you had GWs?

Transitional question	Perception of illness	2. What are your views on this disease (in comparison to other STDs, for instance)? *Cues: Cause, consequences, treatment options, time to cure and knowledge about HPV* 3. Does the fact that it is caused by HPV influence your views on the disease?

Key questions	Effects of GWs on QoL	4. How has your life been affected by having GWs? a. Physical effects b. The course of treatment *Cues: Pain, embarrassment, worries about treatment effectiveness, duration and, knowledge, doctor-patient communication, practical aspects* c. Effects on work and studies *Cues: Concerns about stigma, ability to work/study, sick leave, contagion, social avoidance* d. Social relations *Cues: Concerns about stigma, contagion, avoidance, social isolation* e. We initially wanted to base this study on a group interview, but the MSM we talked to were very reluctant to participate in this and would only participate in an individual interview. Could you explain why this might be? f. Love life *Cues: Worries about present or future partners, infidelity, contagion, conflicts about infidelity or disrupted sex life, fear of rejection and disapproval* g. Sex life *Cues: Desire/lust, initiative, pleasure, spontaneity, avoidance, low self esteem, negative body perception, fear of rejection, lack of sexual ability* h. Psychological effects *Cues: Guilt, shame, anger, worries about the future, depression, fear, negative self perception, identity, insecurity, disease phobia* 5. Has your quality of life changed since you were first diagnosed with GWs, and if yes, how?

Closing questions		6. Do you feel you have sufficient knowledge about GWs? 7. Is there anything you think we ought to have discussed but did not?

GWs, genital warts

The six participants were recruited from the Venereal Diseases Clinic at the Bispebjerg Hospital in Copenhagen, Denmark, and through homosexual media. Patients were eligible if they had suffered from GWs for at least three months and had no serious co-morbidity or other STDs. Patients who fulfilled the inclusion criteria were informed orally at the clinic about the study by the consulting physician, who also gave them a study information sheet. Patients willing to participate contacted one of the authors, GLM, who acted as an independent researcher. The study did not require ethics committee approval in Denmark and all participants gave written informed consent. No personal information about the participants was collected or communicated from the Venereal Diseases Clinic to the authors or other people involved in the study. The participants' anonymity was ensured throughout the study.

Interviews were conducted by GLM in small conference rooms at a hotel or in the participants' homes, according to their preference. The purpose of the study (to obtain comprehensive knowledge about MSM patients' experiences with and perceptions of GWs) was explained. Patients were interviewed using a semi- and funnel-structured interview guide (Table 1), beginning with questions about the participant's perception of GWs: the causes, its management and timeline. Questions then focused on the personal consequences of having GWs, including the effects on the patients' self-esteem, social and love life. Participants were asked about the reasons for their reluctance to participate in a group interview. Questions were open-ended to ensure an exploratory approach and capture as many perspectives as possible [36,37].

Interviews were transcribed verbatim and analysed using a discourse analytical approach to the relations between language and socio-cultural construction of meaning [38]. After coding and classifying data into the main patterns of meaning, a comparative analysis was carried out identifying how the QoL of MSM was affected by having GWs and how this differed from heterosexual patients with GWs. All methodological and analytical steps were discussed and alternative interpretations sought with an independent anthropologist. Disagreements were solved using Spradley's process of resolution of qualitative data [37].

## Results

Six patients, aged between 31 and 59 years participated, and all but one were single. Four participants had intra-anal GWs, five had perianal GWs and one had penile GWs. One participant had had perianal GWs for only three months. The other participants had had GWs for 3 to 10 years, some with periods of remission (Table 2).

**Table 2:** Participant characteristics

	Age (years)	Marital status	Years since first occurrence of GWs	Recurrences	GW location
1	31	Single	3-4	Several (maximum 2 month clearances)	Anal and perianal

2	33	Single	8	Several (short periods of clearance)	Anal and perianal

3	47	Single	9-10	4	Penile and anal

4	31	Single	4-5	1 (1-1.5 year clearance)	Anal

5	51	Single	4-5	3 (maximum 2-3 month clearances)	Perianal

6	59	Married (same sex)	8	None	Perianal

### The perception of GWs among MSM

The participants considered GWs as a repulsive and stigmatising STD that they did not know much about before they themselves were affected. All patients considered that treatment options were limited in terms of effectiveness, duration and prevention of recurrence, and had underestimated the effects of the disease on their psycho-sexual and social QoL. MSM felt frustrated throughout the course of treatment and had a strong need for information about HPV infections and treatment options - including the effects of the HPV vaccine which two participants had already received with the hope of increasing the efficacy of their GW treatment.

In particular, participants said they had substantial needs for information about the relation between GWs and anal cancer, with several expressing worry about the risk of developing anal cancer. In addition to the psycho-sexual and social consequences of GWs, fear of cancer dominated their perception of the seriousness of GWs. These concerns were highly dependent on their doctors' communication and the information they provided about the disease.

"When I read that, I thought 'Oh, my God!'. I had no idea what it was all about! I don't understand whether it's infectious when you just touch each other (anywhere) or if the virus is only local. I don't think that's clear from the information provided. But I could read - and that's when I got all worked up - that anal cancer and God knows what can develop!"(31-year-old MSM)

### GWs in the social context

The participants in this study feared the potential impact that GWs could have on their status in the social group to which many felt a strong sense of belonging. Several participants described the homosexual *scene *as the place where they fitted in and where they were accepted for who they are. Commonly, *the scene *was both the primary social framework for the participants and the starting point for finding a steady or occasional partner. At the same time, *the scene *was depicted as a 'village' where gossip was widespread, and bullying and victimisation were not uncommon. As one participant put it:

"If you are heterosexual, the problem of GWs mainly involves your relationship with your partner, but if you are gay, you also worry about rumours spreading in a minority culture where everybody knows each other and a negative reputation creates barriers to meeting other men and to being accepted." (59-year-old MSM)

The participants in this study frequently compared GWs with HIV. For instance, some said that while GWs were not as serious as HIV, having GWs corresponded to being HIV-positive in its social and sexual consequences. Some said they considered GWs to be more serious because using condoms does not entirely protect against HPV infection, or that GWs had a more restrictive influence on their sex and love life because they were potentially visible.

*"I just know that if I tell anyone, it will be passed on to the others. The rumour will spread and I'll simply be excluded. I'll be branded. I feel branded. I can draw so many parallels to someone who's HIV-positive. That's why I can understand someone who's HIV-positive who won't disclose his status when he is with someone. I can't blame them. It's like HIV 'you don't get infected, you let yourself be infected'. It's the same thing with the GWs, well, I let myself be infected, and it's my own fault! I know it's not possible to protect yourself 100% from GWs but that thought is stuck at the back of my head.... Of course, it's not that GWs are more serious than HIV, not at all, but I understand someone saying that because it is very visible *(this participant had penile GWs), it's very inhibiting and you can't do anything about it. Someone who's HIV-positive can just use a condom and practise safe sex. That's not b***** possible with GWs!"

GLM: "What exactly do you mean by being branded?"

"Well, you might as well deport me to a deserted island. That's how I feel. Because nobody will want anything to do with you at all. You'll be a walking infected abscess and nobody wants to having anything to do with you" (47-year-old MSM)

According to the participants, fear of rejection was the reason given for not wanting to participate in a group interview where there would be a risk of meeting others from *the scene*. One participant had become so sexually insecure after developing GWs that he had entirely withdrawn from *the scene *and thus also its broader social advantages. Another participant had lost his confidence in relationships with other people in general. Participants whose GWs or scarring were visible to potential sexual partners seemed to have experienced the most negative affects on their social lives. They said they feared a sexual *and *social exclusion from *the scene *because their condition would be talked about and this increased the negative psychological effects of the disease.

### The effects of GWs on participants' sex and love lives

According to some participants, the reluctance to participate in a group interview was ultimately about the risk of not finding sex and love:

*"You're exposed to so many external stressful issues - including the society, the family, that homosexuality is not accepted, lack of rights... all those things: girls that you may have dated at the beginning and with whom it didn't work out. It would be weird if you didn't react by becoming sensitive. That's why it's a small scene with a lot of sensitive people. So the reason that you can't talk about it *(GWs) *in a group interview is related to two things: that it's a small scene and that people are sensitive -they are sensitive about being rejected again. Essentially, it's all about love. When people won't participate, it's because they're afraid of not experiencing love because of what will be said in the scene. Because, when I meet people - that applies to one-night-stands or if I've been at the park - it applies to everybody: what people really want is to be cared for. They want to be loved. It's quite simple, actually. But because they've had one downfall after another, bad experiences, families not recognising them, they don't know how love works. They don't know how to receive love. And then quickly, you'll be sorted out. It's also a scene where you can't have children. It goes fast, if things don't just work, you move on!" (31-year-old MSM)*

The location of the GWs and the participants' preferences for receptive or insertive anal sex had an important impact on whether they had any sexual encounters. The decisive factor was whether the GWs or the scarring were visible to the sexual partner and whether the GWs influenced the possibilities of having a sex life. The two participants who had perianal GWs and who no longer practiced receptive anal sex did not have to reveal their GWs, so they could still have sex.

For the other participants, their sex lives were ruined due to fear of infecting others, worries about pain, about disclosure and rejection. Several participants had stopped even trying to find a partner or to have sex, thus reducing their chances of developing a steady relationship from a casual sexual contact. One participant, who still had some occasional sexual encounters, was so preoccupied with hiding the disease during intercourse that it often turned out to be tense and awkward. Most participants had stopped looking for a steady relationship because they were scared about having to reveal their disease. Some had been in relationships that ended because of the devastating effects of the GWs on their sex life. With one exception, all the participants said that having GWs had an extremely negative impact on their sex and love lives.

### The physical and psychological effects of having GWs

The main physical effects of having GWs were related to treatment which often caused pain, soreness and ulcerations. For one participant, prolonged treatment had ruined his ano-genital mucous membranes as well as his anal sphincter. He could not have sex or bowel movements without pain and bleeding. Another participant had stopped treatment for long periods because treatment was much worse than the symptoms of the GWs.

The more the participants' sex and love lives were affected by the GWs, the worse the negative impact on their psychological well-being. In particular, the participants whose sex and love lives had come to a standstill described themselves as being socially and sexually insecure and as loathing their own bodies. Just like heterosexual patients with GWs, the participants worried about the development of their GWs, were often depressed and frustrated with the disease and felt alone in their suffering.

Three participants had had nervous breakdowns which they attributed, to varying degrees, to their GWs, and for which they had subsequently received psychological treatment. According to these participants the disease had ruined their self-esteem and made them feel like 'unclean infected disease carriers'. One 33-year-old participant who had had GWs for eight years felt that he would have been a different person without this disease which had affected him profoundly during his formative years.

*"I recently finished my studies, but they *[GWs] *affect my work life just as they did my studies. It's still as if I don't rise to the occasion. I'm tied up. If I was free from them *[GWs]*, I think I'd also feel freer in my relationships with others, that I could stand by myself and do things more whole-heartedly. I don't really dare to stand out or be noticed. That's due to the GWs, I think. I feel like people think that I'm not really present. That affects people and makes them uncertain of me. I don't show myself and they can feel that there is something wrong. It's hard because I am not 100% there, because I'm still 'disconnected' from here (points to his chest) downwards. I don't think you can be entirely present if your body is not with you... I've got like two jobs but they've got nothing to do with my career. I kind of dread that a bit. I finished studying five months ago and got straight A's so that's good. But it's like I don't dare to go out there and sell myself and that's what I have to do in my line of business. You have to sell yourself and I can't do that" (33-year-old MSM)*.

## Discussion

This study, based on interviews with a small number of participants, is the first to use qualitative methodology to examine in depth the disease experiences of MSM with GWs. Qualitative research methodology allows for an analytical, but not a statistical generalisation of results. This is both a strength and a limitation. In many respects, the results of this study are very similar to those of our study in heterosexual patients with GWs [1] as well as those identified in the literature [3-17]. While this validates the results from the present study, we also identified information about the perspectives of MSM with GWs which need to be confirmed in a larger study.

The method of recruitment may have introduced a selection bias because patients with more negative disease experiences may be more likely to volunteer to participate in this type of study. Nonetheless, we considered participants to be representative of Danish MSM with GWs in terms of the severity of their symptoms, while the social mechanisms of inclusion and exclusion within the homosexual minority culture appear similar to those described in a recent American study in HIV-positive MSM [39].

The significance of any disease is acquired in the context of a particular socio-cultural framework [35]. In the case of GWs in MSM in our study, this includes a broader Western concept of STDs and homosexuality, the Danish society and the homosexual *scene *as a self-defined minority culture. Our results showed that belonging to a homosexual minority group seems to have significant consequences for MSM who have GWs. Two social exclusion mechanisms may reinforce each other when MSM have GWs: in addition to feeling marginalized as a homosexual in a predominantly heterosexual (external) society, MSM fear stigmatisation internally in the homosexual *scene*. Many participants compared their experience of GWs with that of being HIV positive, although this is generally considered to be a far more serious disease. This association was explained by the psycho-sexual and social consequences of having GWs, which the participants put on the same footing as the experiences of homosexual men who are HIV-positive. The US study in MSM showed that stigmatisation of men who were HIV positive in the homosexual scene led to worry, loneliness, depression and avoidance strategies [39]. In addition, these participants had had GWs for a longer period of time than the heterosexuals in the previous study and this added to the negative psychological consequences for them.

Most of the participants in this study said that rejection from the homosexual *scene *would mean a loss of belonging to their significant social group and reduced chances of finding sex and love. Having GWs thus not only affected the participants' sex life, it could also have far-reaching social and psychological consequences. Self-stigmatization, anticipatory stigma and actual experience of stigma or discrimination were thus closely interrelated and worked together in shaping these participants experiences with GWs. In addition, their treatment was frequently painful, with a timeframe which was mostly longer than for the heterosexual participants in the previous study [1].

This study showed that, just like women with GWs fearing the development of cervical cancer when they learnt about HPV, MSM also worried about anal cancer when they were aware of HPV. Both heterosexual and homosexual patients felt that GWs were not considered to be as serious as other potentially fatal STDs by health care professionals and that this lead to a gap between health care professionals perspective and their own worries about GWs. These participants wanted a better understanding of the issues and questions that patients with GWs face. Knowledge about specific minority cultural aspects, including the needs of MSM for clear oral and written information about HPV and anal cancer, should guide patient communication with MSM.

## Conclusions

The prevalence of anal and multiple HPV type infections among MSM are relatively high [23]. Preliminary data from a randomised clinical trial in men have shown that quadrivalent HPV vaccination is efficacious in preventing HPV infection and related diseases in men [40,41]. In a recent review of studies assessing the acceptance of male HPV vaccination, it was reported that the acceptability of HPV vaccination was high among men informed about the direct benefits for men and when they received doctors' recommendations [42-44]. It is important that MSM are well informed about all aspects of GWs, with the aim of alleviating both the psychological distress associated with the disease and to optimise preventive efforts and safe sexual behaviour.

## Competing interests

GLM received a research grant (including the article processing charge) for this study from Sanofi Pasteur MSD. The study was conceived and the protocol was developed independently of this funding. GLM has previously received research grants from Sanofi Pasteur MSD for other studies of HPV related diseases and HPV vaccination as well as fees for orally presenting the results. HKL received financial support from Sanofi Pasteur MSD to participate in a medical conference in 2007.

## Authors' contributions

GLM conceived the study and its design, carried out the data collection, analysed and interpreted the data and drafted the manuscript. HKL participated in the inclusion of patients, drafted the medical parts of the manuscript and revised the manuscript critically for important intellectual content. Both authors have read and approved the final manuscript.

## Author's information

GLM has a MA in social anthropology and is specialised in medical anthropology. She currently works as a research consultant at AnthroConsult specialised in qualitative patient studies.
